# Supplementary figures and images for: Cryptosporidium exports a mucin glycoprotein into the microvilli of intestinal epithelium
Source: Virulence. 2025 Sep 2;16(1):2553780. doi: 10.1080/21505594.2025.2553780 (PMC12407990; doi:10.1080/21505594.2025.2553780)

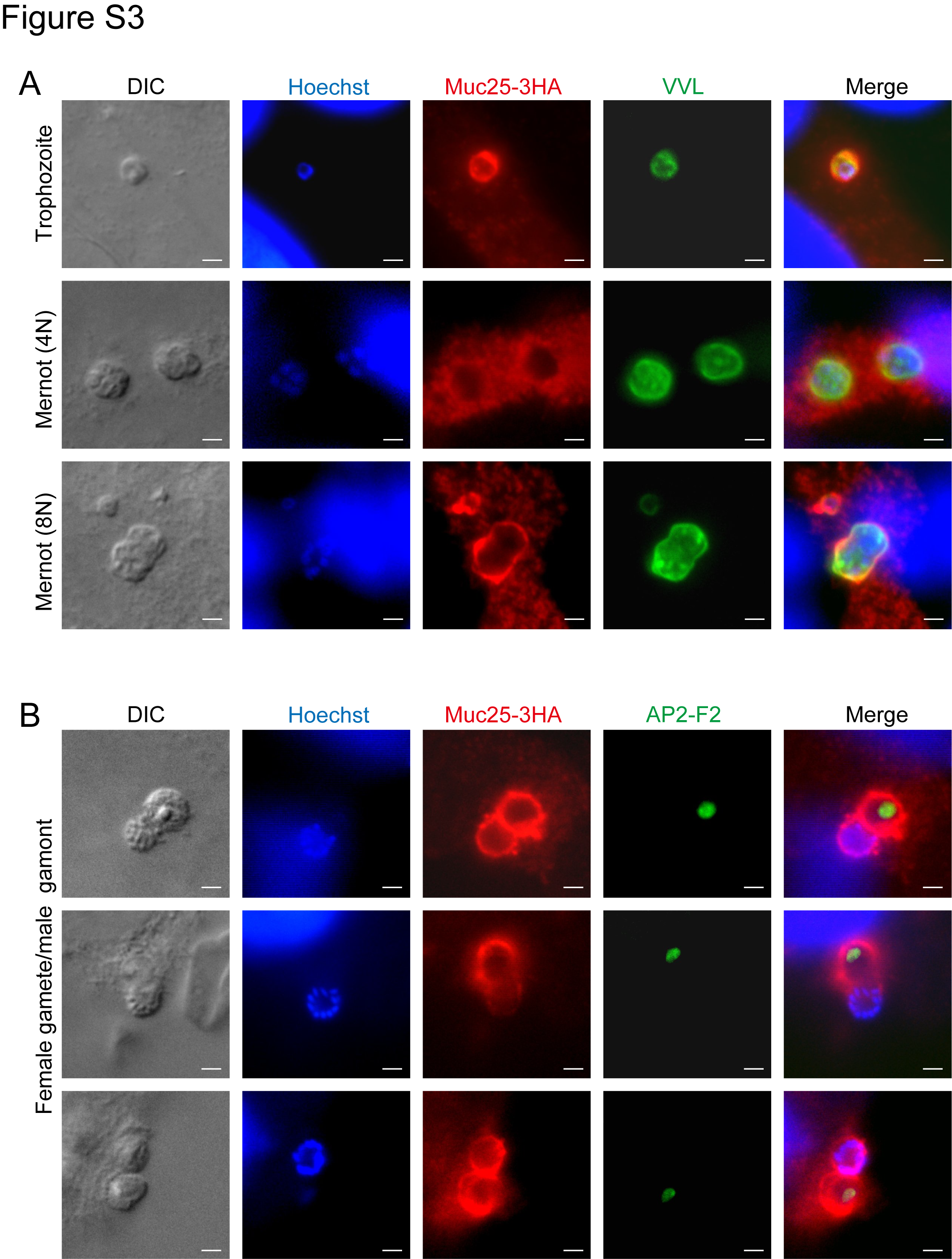

Supplement: Figure S3.tif [file KVIR_A_2553780_SM2340.tif]

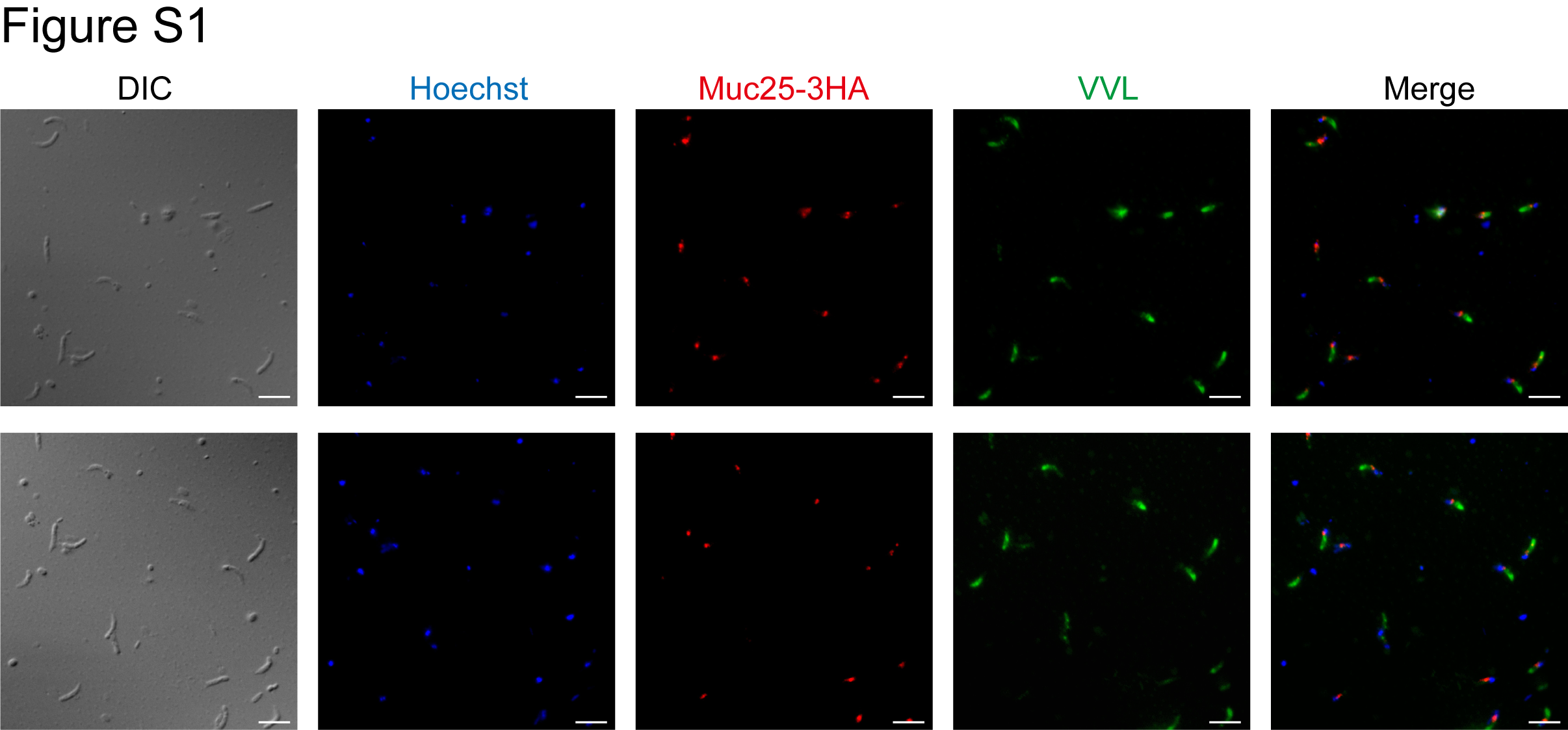

Supplement: Figure S1.tif [file KVIR_A_2553780_SM2339.tif]

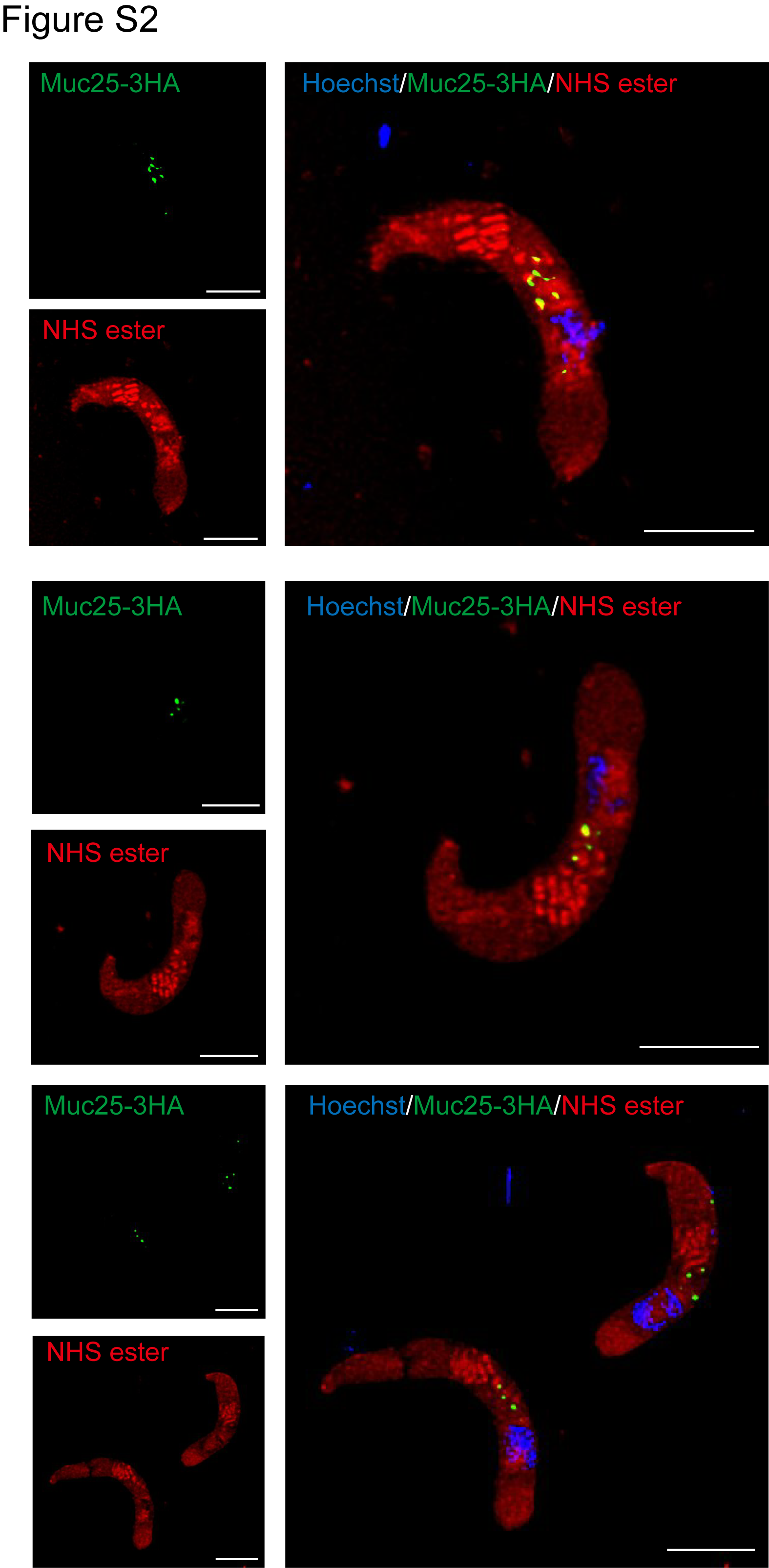

Supplement: Figure S2.tif [file KVIR_A_2553780_SM2336.tif]
