# Supplementary material for: Cryptosporidium exports a mucin glycoprotein into the microvilli of intestinal epithelium
Source: Virulence. 2025 Sep 2;16(1):2553780. doi: 10.1080/21505594.2025.2553780 (PMC12407990; doi:10.1080/21505594.2025.2553780)
Supplement: Muc25_Revised manuscript_clean copy_for editor_20250603.docx [file KVIR_A_2553780_SM2337.docx]

***Cryptosporidium* exports a mucin glycoprotein into the microvilli of intestinal epithelium**

Rui Xu ^1,#^, Jieping Li ^1,#^, Wenjie Wu ^1^, Zuwei Yang ^1^, Shengchen Zhang ^1^, Fuxian Yang ^1^, Tianyi Hou ^1^, Yaqiong Guo ^1^, Yaoyu Feng ^1^, Lihua Xiao ^1,^*, Na Li ^1,^*

^1^ State Key Laboratory for Animal Disease Control and Prevention, Center for Emerging and Zoonotic Diseases, College of Veterinary Medicine, South China Agricultural University, Guangzhou 510642, China.

^#^ These authors contributed equally.

* Contact E-mail: [lxiao1961@gmail.com](mailto:lxiao1961@gmail.com) (LX), [nli@scau.edu.cn](mailto:nli@scau.edu.cn) (NL)

**Abstract**

*Cryptosporidium* causes severe diarrhea in humans and animals. Mucin-like glycoproteins play a critical role in parasite attachment and invasion and therefore serve as potential protective antigens against reinfection. Muc25 is a highly polymorphic mucin that has been associated with differences in host infectivity in comparative genomic analyses. To study the function of Muc25, we determined its localization and secretion in *Cryptosporidium parvum* by genome editing. Endogenous gene tagging revealed that Muc25 is stored in small granules of sporozoites and secreted into host microvilli after invasion. Deletion of the signal peptide affected Muc25 localization and secretion, as the Muc25ΔSP protein was localized to the membrane in sporozoites and remained within intracellular life stages. In addition, a *Muc25* knockout strain (*ΔMuc25*) was easily generated, indicating that Muc25 is not critical for parasite survival. However, the *ΔMuc25* strain showed reduced growth in HCT-8 cells, and the survival time was prolonged in GKO mice infected with *ΔMuc25* compared to those infected with Muc25-3HA. Transcriptome analysis revealed that *ΔMuc25* parasites caused less damage to host cells *in vitro* than Muc25-3HA parasites. Taken together, these data provide evidence for the export of a *Cryptosporidium* protein to host microvilli and demonstrate that such manipulation of the host cell response may be involved in parasite pathogenesis.

**Keywords:**

*Cryptosporidium*, glycoprotein, mucin, effector, parasite-host interaction, microvillus

**Introduction**

Cryptosporidiosis, a disease caused by the intracellular pathogen *Cryptosporidium,* is one of the leading causes of moderate to severe diarrhea in humans and animals [1,2]. Importantly, cryptosporidiosis in children can increase the risk of malnutrition, stunted growth, and even death in resource-poor countries [2,3]. There is no effective treatment for cryptosporidiosis. Nitazoxanide, the only FDA-approved drug, has low efficacy in malnourished children and immunocompromised individuals [4]. A vaccine would be the most impactful tool to reduce pediatric cryptosporidiosis. However, the development of a vaccine to prevent human cryptosporidiosis remains in the experimental stage [5]. More basic research is needed to elucidate the mechanism of host-parasite interaction in *Cryptosporidium*.

*Cryptosporidium* forms an intracellular niche at the brush border of intestinal epithelial cells. The parasite resides between the outer and inner membranes of host cells at the apical surface of the epithelium, where it undergoes a complex life cycle. During infection, the parasite rapidly remodels the host cell including formation of a parasitophorous vacuole (PV) and establishment of a complex structure, the feeder organelle, at the host-parasite interface [6-8]. Prior to oocyst shedding, *Cryptosporidium* undergoes multiple rounds of invasion and egress, with each replication taking approximately 12 h [9]. At the same time, the small intestinal epithelium undergoes rapid regeneration and turnover [10]. In addition, *Cryptosporidium* induces remarkable pathological changes in the host intestine, including microvillus blunting and elongation, crypt hyperplasia, and epithelial dysplasia [10-12]. Therefore, *Cryptosporidium* infection has a significant impact on the structure of the intestinal epithelial cells, although the mechanism of this damage remains unclear.

*Cryptosporidium* employs mucin-like glycoproteins to participate in the initial attachment and invasion of the parasite [13]. Mucins are glycoproteins composed of 20% - 55% amino acids with serine, threonine and proline residues [14,15]. These residues are extensively O-linked glycosylated, with 40% - 80% of the molecular weight of these proteins being O-linked carbohydrates [16]. *Cryptosporidium parvum* genome contains 29 genes encoding mucins [17,18]. Furthermore, the number of genes encoding mucin-like glycoproteins differs between *Cryptosporidium* species, contributing to the tissue tropism observed between the intestinal and gastric species [19]. Several mucin-like glycoproteins have been identified as playing an important role in parasite attachment and invasion, such as GP900 [14,20], GP60 (GP40/15) [21,22], Muc4 and Muc5 [23], and AGP1 and AGP2 [24]. Some have been identified as playing a role in protective immunity against reinfection in humans, including GP900, GP60, CP23 and Muc20 (cgd8_700) [25], suggesting that mucins may be vaccine candidates.

Previous studies identified mucin 25 (Muc25) as a small glycoprotein containing 72 O-type glycosylation sites and 2 N-type glycosylation sites [19]. Comparative genomic analysis revealed that the *Muc25* gene (cgd6_40) is highly polymorphic among *C. parvum* subtypes, making it a commonly used subtyping marker (MSC6-7) for this species [26,27]. Results from population genetic and evolutionary genomic studies suggest that Muc25 is involved in the adaptive evolution of different *C. parvum* populations [28]. Therefore, Muc25 may play an important role in *Cryptosporidium* biology. However, this theory requires experimental validation.

In the present study, we localized the expression of Muc25 protein, followed its fate using endogenous gene tagging, and evaluated its essentiality for growth and involvement in pathogenesis using gene deletion. Our results indicate that Muc25 is stored in small granules in sporozoites and exported to the host cell microvilli soon after invasion. Knockout of the *Muc25* gene in *C. parvum* reduced parasite growth and host cell damage *in vitro* and prolonged the survival of infected mice *in vivo*. These observations expand the repertoire of *C. parvum* effectors by identifying a parasite protein exported to host microvilli.

**Methods**

**Animal studies**

Animal studies were approved by the Institutional Animal Care and Use Committee of South China Agricultural University under the protocol of 2021c094. *Ifng^-/-^* mice (referred to as GKO) purchased from the Institute of Laboratory Animal Science (Chinese Academy of Medical Sciences) were bred in-house at the Laboratory Animal Center of South China Agricultural University and were separated by sex after weaning. ICR mice used from immunization were purchased from the Guangdong Province Medical Experimental Animal Center. Mice were housed individually in isolators (one mouse per cage) in a specific pathogen-free facility and fed with sterile feed and filtered tap water. During the experiments, mice with more than 20% body weight loss or appearing debilitated were considered moribund and euthanized. Mice were euthanized in an automated CO₂ euthanasia chamber, which is acceptable under the American Veterinary Medical Association's guidelines on the euthanasia of animals.

**Cell culture**

Human ileocecal adenocarcinoma (HCT-8) cells (ATCC CCL-244) were cultured in RPMI 1640 medium (Gibco, Grand Island, NY, USA) supplemented with 10% fetal bovine serum (ExCell Bio, Suzhou, China) at 37°C in a 5% CO_2_ incubator.

**Parasite preparation**

*Cryptosporidium parvum* IIdA20G1 (HLJ isolate) was maintained by repeated passages in GKO mice and purified from fecal material [29]. Purified oocysts were stored in phosphate-buffered saline (PBS) plus 1 mg/ml ampicillin, 1 mg/ml streptomycin, and 0.5 mg/ml vancomycin at 4°C for up to six months after fecal collection. Prior to infection of cell culture or mice, oocysts were treated with 10% bleached solution (the final concentration of sodium hypochlorite was 0.52%) on ice for 10 min and washed three times with PBS. Bleached oocysts were resuspended in PBS plus 1% bovine serum albumin (BSA) and stored at 4°C for up to one month. For experiments requiring sporozoites, bleached oocysts were excysted in 0.75% sodium taurocholate diluted in PBS at 37°C for 1 h.

**Sequence analysis**

The nucleic acid sequences of Muc25 in *C. parvum*, *C. hominis*, *C. tyzzeri* and *C. mortiferum* were extracted from CryptoDB (<https://cryptodb.org/cryptodb/app>) and NCBI (<https://www.ncbi.nlm.nih.gov/>). A maximum likelihood phylogenetic tree was constructed with 1,000 replicates of bootstrapping. Glycosylation analysis was performed using YinOYang 1.2 (https://services.healthtech.dtu.dk/services/YinOYang-1.2/) and NetNGlyc 1.0 (https://services.healthtech.dtu.dk/services/NetNGlyc-1.0/).

**Primers and plasmids**

All primers were synthesized by Sangon Biotech (Shanghai, China) and are listed in the Table S1, together with all plasmids used and generated in this study.

**Construction of CRISPR/Cas9 plasmids**

To generate the tagging plasmid, a 5’ homology fragment from the 543 bp upstream before the stop codon of *Muc25* and a 3’ homology fragment from the 501 bp downstream after the stop codon of *Muc25* were amplified from *C. parvum* genomic DNA by PCR. The 3HA-Nluc-P2A-neo reporter and the pUC19 plasmid backbone were amplified from the pINS1-3HA-Nluc-P2A-neo plasmid [30]. The tagging plasmid was then generated by Gibson assembly of the components described above. To generate the signal peptide knockout plasmid, 5’ homology fragments from the 891 bp 5’UTR of *Muc25* and 468 bp of the signal peptide deleted *Muc25* ORF, and a 3’ homology fragment from the 501 bp 3’UTR of *Muc25* were amplified from *C. parvum* genomic DNA by PCR. The signal peptide knockout plasmid was then generated by Gibson assembly of these components, the 3HA-Nluc-P2A-neo reporter and the pUC19 plasmid backbone as described above. To generate the knockout plasmid, a 5’ homology fragment from the 501 bp 5’UTR of *Muc25* and a 3’ homology fragment from the 493 bp 3’UTR of *Muc25* were amplified from *C. parvum* genomic DNA by PCR. The Nluc-P2A-neo cassette and the pUC19 plasmid backbone were amplified from the pINS1-3HA-Nluc-P2A-neo plasmid. The knockout plasmid was then generated by Gibson assembly of the components described above.

To generate the Cas9 plasmid, a single guide RNA (sgRNA) targeting the 3’ end, 5’ end, or signal peptide of the *Muc25* gene was designed using the eukaryotic pathogen CRISPR guide RNA/DNA design tool (<http://grna.ctegd.uga.edu>). The Cas9 plasmid was generated using the designed sgRNA and a linear Cas9 plasmid amplified from pACT:Cas9-GFP, U6:sgINS1 [30] by Gibson assembly.

**Selection and amplification of transgenic *C. parvum* in mice**

For transfection, sporozoites were suspended in SF buffer (Lonza, Basel, Switzerland), mixed with 50 μg tagging plasmid and 30 μg Cas9 plasmid, and electroporated on an AMAXA 4D-Nucleofector system (Lonza) using the EH100 program. Electroporated sporozoites were transferred to cold PBS at a 1:1 ratio and placed on ice prior to infection. Each GKO mouse was first gavaged with 200 μl of 8% sodium bicarbonate. Five min later, the mouse was gavaged with 100 μl of electroporated sporozoites. All mice received 16 g/l paromomycin drinking water 1 day post infection (dpi) based on previously published protocol [31]. Fecal pellets were collected starting at 5 dpi and stored at 4°C for luciferase assay, PCR analysis and oocyst purification. For amplification of transgenic parasites, GKO mice were gavaged with 1 × 10^4^ oocysts/mouse and treated with 16 g/l paromomycin in drinking water throughout the experiment. Fecal pellets were collected daily once the mice began shedding oocysts.

**PCR analysis**

DNA was extracted from fecal materials using the Fast DNA Spin Kit for Soil (MP Biomedicals, Irvine, CA, USA). To verify the proper integration of the transgenic strains, PCR was performed using the fecal DNA, EasyTaq PCR SuperMix (Transgen, Beijing, China), and the primers listed in the Table S1. PCR gels were viewed on the UVP ChemStudio imaging system (Analytik Jena, Jena, Germany) and imaged using VisionWorks software (Analytik Jena).

**Whole genomic sequencing**

DNA samples were extracted from purified Muc25ΔSP and *ΔMuc25* oocysts after five freeze-thaw cycles using a QIAmp DNA Mini Kit (Qiagen, Hilden, Germany). Whole genome amplification was performed on the DNA using the REPLI-g Midi Kit (Qiagen). Products were sequenced on an Illumina HiSeq 2500 (Illumina, San Diego, CA, USA) using the 150 bp paired-end approach. After trimming for adaptor sequences and poor sequence quality (<phred score less than 25), the sequence reads were mapped to the reference genome of IIdA20G1-HLJ using BWA-MEM for verification of the complete deletion of the target sequences using Integrative Genomics Viewer.

**Luciferase assay**

Luminescence values were measured using the Nano-Glo luciferase assay kit (Promega, Madison, WI, USA). To monitor parasite shedding in mice, fecal pellets were collected in a 1.7 ml tube, ground, and mixed with ten 3-mm glass beads (Thermo Fisher Scientific, Carlsbad, CA, USA) and 1 ml fecal lysis buffer (50 mM Tris pH 7.6, 2 mM DTT, 2 mM EDTA pH 8.0, 10% glycerol, 1% Triton X-100 prepared in ddH_2_O). Samples were homogenized with the FastPrep bead beating grinder (MP Biomedicals) at 6.0 m/sec for 45 sec and centrifuged at 19,000  ×  *g* for 1 min to pellet debris. 50 μl supernatant was transferred to one well of a 96-well white plate (Thermo Fisher Scientific) and mixed with 50 μl Nano-Glo luciferase buffer with substrate, incubating for 3 min at room temperature (RT). Luminescence values were read on a Synergy HT multimode reader (BioTek, Winooski, VT, USA).

To measure parasite burden in cells, HCT-8 cells infected with *C. parvum* in a 24-well plate were washed three times in PBS, added 100 μl of Nano-Glo luciferase buffer, and incubated for 10 min at 37°C. Samples were scraped from wells and transferred to one well of a 96-well white plate, added 2 μl Nano-Glo luciferase substrate, and incubated for 3 min at RT. Luminescence values were read on a Synergy HT multimode reader (BioTek).

**Generation of polyclonal antiserum against *C. parvum* AP2-F2**

Recombinant *C. parvum* AP2-F2 (encoded by cgd2_3490) was expressed in a prokaryotic expression system to produce the anti-AP2-F2 polyclonal antibody used to stain the nucleus of female gamonts. Two DNA fragments (982-1227 bp and 2125-3222 bp) of the cgd2_3490 gene were amplified and linked as a fused segment. It was then cloned into the pE-SUMO vector and expressed as a fused protein in *E. coli* BL21 (DE3) cells. The recombinant protein was purified using Ni-Sepharose (General Electric, Boston, MA, USA) and used to immunize five ICR mice with Freund’s complete and incomplete adjuvants (Sigma-Aldrich). Sera were collected 14 days after the last immunization, and the preimmunize sera were used as control sera.

**Immunofluorescence microscopy**

For extracellular parasite imaging, sporozoites were placed on slides, fixed with 4% formaldehyde-PBS for 15 min and permeabilized with 0.5% TritonX-100 in PBS for 10 min. For intracellular parasite imaging, HCT-8 cells were plated on 12-mm diameter glass coverslips in a 24-well plate and grown to confluence. Monolayers were infected with sporozoites and cultured for a specific time. Cells were fixed with 4% formaldehyde-PBS for 15 min and permeabilized with 0.5% Triton X-100 in PBS for 10 min. Cells were incubated with rabbit anti-HA primary antibodies (CST, Danvers, MA, USA) diluted 1:1000 in 1% BSA-PBS at 4°C for overnight, washed three times in PBS, and incubated with *Vicia Villosa* Lectin (VVL) fluorescein (Vector, Newark, NJ, USA) diluted 1:500 and Alexa Fluor-conjugated secondary antibodies (Thermo Fisher Scientific) diluted 1:1000 in 1% BSA-PBS for 60 min. To identify sexual stages, HCT-8 cells infected with sporozoites were harvested at 48 h post-infection (hpi), fixed, and permeabilized as described above. The cells were then incubated with rabbit anti-HA primary antibodies (CST) diluted 1:1000 and mouse anti-AP2-F2 serum diluted 1:200 in 1% BSA-PBS at 4°C for overnight, washed three times in PBS, and incubated with Alexa Fluor-conjugated secondary antibodies (Thermo Fisher Scientific) diluted 1:1000 in 1% BSA-PBS for 60 min. Nuclear DNA was stained with Hoechst (Thermo Fisher Scientific) diluted 1:1000 in PBS for 20 min. Finally, the slides and coverslips were washed three times in PBS and mounted with Antifade mounting medium (Beyotime, Shanghai, China). Images were captured using a BX53 biological microscope equipped with a 100× oil objective lens (Olympus, Tokyo, Japan) and processed using ImageJ (https://fiji.sc/).

**Ultrastructure expansion microscopy (U-ExM)**

U-ExM was performed as previously described [32]. Briefly, excysted sporozoites were placed on poly-D-lysine-coated coverslips, incubated for 20 min, and fixed with 4% formaldehyde-PBS for 5 min. Samples were secondarily fixed with acrylamide and formaldehyde solutions to prevent cross-linking prior to gel embedding. The resulting gel was transferred to a denaturing buffer, denatured at 95°C for 1 h, and expanded in water overnight to approximately 4 times its original size. The gel was then shrunk in PBS and stained with primary antibody (rat anti-HA diluted 1:200 in 2% BSA) overnight at 4°C. After three washes in PBS, the gel was incubated with Alexa Fluor-conjugated secondary antibodies (Thermo Fisher Scientific) diluted 1:200 and Hoechst diluted 1:250 in PBS at 37°C for 3 h. Finally, the gel was incubated with Atto 565 NHS ester (Sigma-Aldrich, St. Louis, MO, USA) at 10 mg/mL in PBS at 37°C for 3.5 h and re-expanded one more time in water. Images were captured on a Stellaris 5 confocal microscope equipped with a 100× oil objective lens (Leica, Wetzlar, Germany) and acquired using LAS X software with a super-resolution lightning system (Leica).

**Immunoelectron microscopy**

GKO mice were gavaged with 1 × 10^4^ oocysts/mouse and treated with 16 g/l paromomycin in drinking water throughout the experiment. At 12 dpi, mice were euthanized, and ileal tissues were harvested. Tissues were fixed in 0.1% glutaraldehyde for 4 h at 4°C, followed by 2% paraformaldehyde at 4°C overnight. Tissues were washed in 0.1 M glycine, dehydrated in ethanol, and embedded in LR White resin (Sigma) at -20°C for 48 h. The embedded samples were sectioned at 70 nm using an EM UC7 ultramicrotome (Leica) and mounted on single-mesh formvar carbon-coated nickel grids. Samples on nickel grids were then blocked and incubated with rabbit anti-HA antibody (CST) diluted 1:20, followed by goat anti-rabbit IgG 10 nm colloidal gold diluted 1:20 (Sigma-Aldrich) and stained with 2% uranium dioxide acetate (SPI Supplies, West Chester, PA, USA) and 10% lead citrate (SPI Supplies). Images were captured on a Talos L120C electron microscope (Thermo Fisher Scientific) operating at 120 kV.

***In vitro* parasite growth assay**

HCT-8 cells were grown to confluence in 24-well plates and infected with 1 × 10^4^ bleached Muc25-HA or *ΔMuc25* oocysts per well. At specific points, cells were washed three times in PBS, and luminescence values were measured as described above.

**Infection studies in mice**

A total of 12 GKO mice, 3-5 weeks old, were randomly divided into 2 groups: the Muc25-HA group and the *ΔMuc25* group. The mice were housed in individual cages throughout the course of the infection studies. Each mouse was gavaged with 1 × 10^3^ transgenic oocysts and received 16 g/l paromomycin drinking water throughout the experiment. Fecal samples were collected at two-day intervals from 0 dpi to 30 dpi and stored at 4°C for luciferase assays. Luminescence values of fecal pellets were measured as described above. Mice were weighed at each sampling and the time of death was recorded to construct a survival curve.

**RNA sequencing**

HCT-8 cells were grown until confluence in a 48-well plate and were infected with 2 × 10^5^ Muc25-3HA or *ΔMuc25* oocysts/well or uninfected as a control. Total RNA was extracted from cells at 12 hpi using Trizol reagent (Thermo Fisher) according to the manufacturer’s instructions. Samples were sent to Genedenovo Biotechnology Co. (Guangdong, China) for RNA sequencing. The RNA libraries were prepared using an Illumina TruSeqTM RNA Sample Prep Kit (Illumina) and sequenced using an Illumina Novaseq 6000 (Illumina). The RNA data were used to identify differentially expressed genes (DEGs) between the Muc25-3HA, *ΔMuc25,* and control groups using the following criteria: absolute fold change > 1.5 and *P* value < 0.05. DEGs were subjected to Kyoto Encyclopedia of Genes and Genomes (KEGG) pathway enrichment analysis and Gene Ontology (GO) term enrichment analysis using the ClusterProfiler package (v4.10.0). A Venn diagram of shared orthologs between groups was drawn using VennPainter (v1.2.0).

**Statistical analysis**

All statistical analyses were performed in GraphPad Prism 9.0. No data was excluded from analyses and *P* value < 0.05 was considered statistically significant. Information on specific statistical tests, technical replicates (n), and *P* values are given in the figure legends.

**Statement of adherence to ARRIVE guidelines**

In this study, we followed the Animal Research: Reporting of *In Vivo* Experiments (ARRIVE) guidelines to ensure the transparency and integrity of our experimental design, execution and reporting. We have adhered to ARRIVE guidelines and uploaded a complete checklist as supplementary files.

**Results**

**Muc25 is a small granule protein of *C. parvum***

Genomic analysis revealed that *Muc25* is a highly polymorphic gene among *Cryptosporidium* spp. Here, we compared the nucleotide sequences of *Muc25* among several closely related *Cryptosporidium* spp. Neighbor-joining phylogeny showed that the *C. parvum* IId *Muc25* clustered together with *C. parvum* IIa *Muc25* in a larger group that contained *C. tyzzeri* *Muc25* but was distant from *Muc25* of the anthroponotic *C. parvum* IIc (**Figure 1A**). Muc25 is a serine-rich protein, similar to other *C. parvum* mucins [14], and contains 72 O-type glycosylation sites and 2 N-type glycosylation sites. Transcriptional analysis revealed that Muc25 maintained relatively high transcript levels in asexual and sexual stages including sporozoites, a transcriptional pattern similar to small granule proteins in previous studies (**Figure 1B**) [22,33].

To investigate the localization of Muc25, we employed the CRISPR/Cas9 gene editing to add a triple hemagglutinin (3HA) tag to the C-terminus of Muc25 and the nanoluciferase (Nluc) and neomycin (neo) cassette after Muc25 (**Figure 1C**). After transfection and selection in GKO mice, PCR analysis of oocysts collected from the mice confirmed that the tagging and selection cassette were correctly inserted into the *Muc25* locus (**Figure 1D**). To investigate the localization of Muc25 in *C. parvum* sporozoites, we performed an immunofluorescence assay (IFA) using anti-HA antibody to stain the Muc25 protein and VVL to stain the sporozoite cytoplasm. Muc25 staining appeared punctate anterior to the nucleus of the sporozoite (**Figure 1E, Figure S1**). We then used ultrastructure expansion microscopy (U-ExM) to localize Muc25 expression, and the images revealed that Muc25 was localized in numerous small vesicles around the nucleus of the sporozoite (**Figure 1F, Figure S2**). This staining pattern was highly consistent with the localization of *C. parvum* small granules observed by U-ExM in previous studies [33], indicating that Muc25 is a small granule protein in *C. parvum* sporozoites.

**Muc25 is exported into host cell microvilli soon after invasion**

To visualize the location of Muc25 in intracellular parasites, we infected HCT-8 cells with the Muc25-3HA strain and examined the localization of the tagged protein at 24 hpi and 48 hpi by IFA using anti-HA antibody to stain the Muc25 protein, VVL to stain the parasitophorous vacuole of intracellular parasite and anti-AP2-F2 to stain the nucleus of female gamete. Asexual stages (trophozoites, meronts and merozoites) have been reported to occur in cell culture from 2 hpi to 36 hpi, followed by predominantly sexual stages (female gametes and male gamonts) at 48 hpi [34]. Muc25 was found to be expressed in all intracellular life stages, both asexual and sexual parasites (**Figure 2A and Figure S3A, B**). Furthermore, HCT-8 cells showed HA staining in the cytosol, suggesting that Muc25 is exported by the parasites into host cells during invasion (**Figure 2A, Figure S3A**). To better understand the timing of export, HCT-8 cells were infected with Muc25-3HA sporozoites, and IFA was performed from 30 min to 2.5 hpi. Initially, HA staining showed a small spot in the parasites, and then, as the trophozoite grew, the size of the HA staining became larger than the early invasion. At 2.5 hpi, Muc25-3HA staining was observed throughout the host cell cytosol, suggesting that it is exported from trophozoites into host cells at 2 hpi (**Figure 2B**). To investigate the localization of the Muc25 protein *in vivo*, we performed transmission immunoelectron microscopy on sectioned mouse intestines infected with the Muc25-3HA strain. Muc25 was detected on the parasitophorous vacuole membrane (PVM) and on the microvilli of host cells (**Figure 2C**). These findings suggest that Muc25 is not injected by the sporozoites during invasion, but it is exported by the trophozoites and reaches the microvilli of host cells.

**Muc25 export is blocked by deletion of the signal peptide**

We further tested whether the signal peptide is involved in Muc25 export. We used CRISPR/Cas9 genome editing to knock out the signal peptide of Muc25 (**Figure 3A**). The transgenic *Muc25ΔSP* strain was isolated from paromomycin-treated GKO mice, and the correct genomic insertion was confirmed by PCR (**Figure 3B**). Mapping of whole genome sequencing reads further confirmed the deletion of the signal peptide sequence (**Figure 3C**). IFA results demonstrated that the knockout of the signal peptide altered the localization of Muc25, with the Muc25ΔSP protein localized to the sporozoite membrane (**Figure 3D**). Furthermore, we infected HCT-8 cells with the Muc25-3HA and *Muc25ΔSP* strains and assessed the localization of the Muc25 protein. When examined by IFA, Muc25ΔSP protein was expressed in the parasite membrane during all intracellular life stages (**Figure 3E**). Taken together, these results suggest that the removal of the signal peptide results in the prevention of Muc25 export with the subsequent accumulation of the protein within the parasite.

**Muc25 is dispensable for parasite growth**

To explore the role of Muc25 in *C. parvum*, we attempted to generate a Muc25 knockout (*ΔMuc25*) strain by replacing the endogenous locus with the Nluc-neo selection cassette (**Figure 4A**). The knockout strain was selected and amplified in paromomycin-treated GKO mice. The deletion of *Muc25* and the correct genomic insertion of the selection marker were validated by diagnostic PCR (**Figure 4B**). Mapping of whole genome sequencing reads further confirmed that the absence of the Muc25 open reading frame in the *ΔMuc25* strain, indicating that Muc25 has been knocked out in the *C. parvum* genome (**Figure 4C**). To characterize the growth of the knockout strain, we performed an *in vitro* infection of HCT-8 cells and an *in vivo* study in GKO mice with *ΔMuc25* and Muc25-3HA parasites. Parasite growth in HCT-8 cells was measured at specific time points using a luciferase assay. The results demonstrated that the *ΔMuc25* strain exhibited a similar growth pattern to the Muc25-3HA strain, while it had reduced growth at 24 hpi and thereafter (**Figure 4D**). However, no difference in oocyst shedding level was observed when comparing the mice infected with the *ΔMuc25* strain to those infected with the Muc25-3HA strain during the course of infection (**Figure 4E**). We also compared the effect of the knockout strain on parasite pathogenicity. The body weight of mice infected with the *ΔMuc25* strain did not differ from that of mice infected with the Muc25-3HA strain (**Figure 4F, G**). However, mice infected with *ΔMuc25* parasites had a longer survival time than those infected with Muc25-3HA parasites (**Figure 4H**).

**Ablation of Muc25 in *C. parvum* reduces host cell damage**

To validate the changes in host cells caused by the Muc25-3HA or *ΔMuc25* strain during the infection, HCT-8 cells were cultured in 24-well plates and infected with1 × 10^4^ Muc25-3HA or *ΔMuc25* oocysts or uninfected as a control. Cells were harvested at 12 hpi and RNA-Seq analysis was performed. Upon analysis of the transcriptome data, Muc25 transcripts were not detected in the *ΔMuc25* samples, further indicating that the *Muc25* gene was knocked out (**Figure 5A**). Differentially expressed genes (DEGs) were identified using the following criteria: at least a 1.5-fold change and *P* < 0.05 between two groups. However, this did not yield any *C. parvum* genes between the Muc25-3HA and *ΔMuc25* groups. In contrast, when the expression of human genes was compared between the *ΔMuc25* and Muc25-3HA groups, 662 DEGs were identified, including 104 upregulated genes and 558 downregulated genes (**Figure 5B**). Further analysis was performed on the Gene Ontology (GO) terms of these DEGs. Here, we showed only the top 5 GO terms for upregulated and downregulated biological process (BP), cellular component (CC), molecular function (MF), and Kyoto Encyclopedia of Genes and Genomes (KEGG) category between the Muc25-3HA or *ΔMuc25* groups (**Figure 5C, D**). For the GO terms associated with epithelial cell-cell adhesion, specifically adherens junction and neutral L-amino acid transmembrane transporter activity, the *ΔMuc25* group showed upregulated transcription compared to the Muc25-3HA group, suggesting that the occurrence of less damage to the cellular junction of host cells in *ΔMuc25*-infected mice (**Figure 5C**). Consistent with this, the GO terms related to TGF-β signaling showed downregulated transcription in the *ΔMuc25* group compared to the Muc25-3HA group (**Figure 5D**). A total of 7 GO terms were found to be downregulated in the *ΔMuc25* group compared to the Muc25-3HA group but upregulated in the Muc25-3HA group compared to the control group, including TGF-β signaling, growth factor binding, sulfur compound binding, and regulation of myeloid cell apoptotic process (**Figure 5E, F**). Taken together, these results indicate that *ΔMuc25* parasites caused less *in vitro* cell damage than the Muc25-3HA parasites.

**Discussion**

Parasite-host interaction has long been known to be important in the infection and pathogenesis of *Cryptosporidium* spp. [35]. Results from previous studies suggest that several mucin-like glycoproteins of *Cryptosporidium* are involved in the initial attachment and invasion of the parasite [21-24]. In this study, we have characterized the newly identified Muc25 of *C. parvum* and demonstrated that it is a small granule protein that is secreted into host cell microvilli during parasite development. Deletion of the Muc25 signal peptide blocks the translocation of Muc25 into host cells. Furthermore, the ablation of the *Muc25* slows parasite growth *in vitro* and attenuates the pathogenicity of a virulent isolate *in vivo*. These results suggest that Muc25 may play a role in *C. parvum* pathogenesis by modifying host cell responses.

Muc25 represents a third family of effector proteins that are exported into host cells. Previously, several ROP and MEDLE proteins of *C. parvum* have been shown to be exported into host cells and to modulate host cell responses. Among them, the rhoptry effector ROP1 has been reported to be injected into the host cells to interact with the cytoskeletal regulator LMO7 [36]. Another effector protein, MEDLE2, has been shown to translocate into the host cell cytosol, triggering ER stress [37]. In the present study, we have shown that Muc25, one of the largest protein families, the mucin glycoproteins, is also secreted into the host cell by *C. parvum*. Other known members of the mucin family of secretory proteins include GP900, GP60, Muc4, and Muc5. While some of these are known as microneme proteins [15,33,38], none of them have been found to be exported into host cells. Here, we find that Muc25 can be translocated into the host microvilli, suggesting that mucins may also be *Cryptosporidium* effectors. This is a novel function for *Cryptosporidium* mucins. Previously, mucins were thought to be mainly involved in parasite-host cell interaction during invasion. For example, GP60 is expressed on the surface of sporozoites, is shed when sporozoites and merozoites glide on host cells and is secreted to the parasite-host cell interface during invasion [22,39]. Another mucin, GP900, is stored in sporozoite micronemes, translocated to the parasite surface during excystation, shed during gliding, and secreted into the extracellular space during parasite development [38]. Probably due to their membrane and immunodominant nature, both GP60 and GP900 have been associated with protection against reinfection in humans [25].

Muc25 is one of the few small granule proteins that have been studied. Apicomplexans release specific effector molecules from secretory organelles, including micronemes, rhoptries, and dense granules [40]. Recently, *C. parvum* has been shown to have a fourth secretory organelle, small granules, which also secrete proteins during invasion [33]. In *Cryptosporidium*, rhoptry proteins may be the key to parasite invasion and trigger actin polymerization [36,41], and microneme proteins are likely involved in parasite adhesion and invasion [38,42]. In addition, dense granule proteins have been demonstrated to be secreted at the parasite-host interface and involved in the formation and function of the parasitophorous vacuole membrane and feeder organelle [33]. To date, there has only been a preliminary study of two small granule proteins, SG1 and SG2, which have been shown to be secreted into the parasite-host interface and parasitophorous vacuole, respectively, during intracellular development [33]. In our study, we provide evidence that Muc25 is an SG protein that is highly expressed at all developmental stages and is secreted into host cells during parasite growth after invasion is already complete. These results suggest that SG proteins, in addition to rhoptry proteins, may also be exported into host cells as *Cryptosporidium* effectors [33,36]. The secretion of both MEDLE2 (at 5.5 hours) and Muc25 (at 2.5 hours) into the host cell cytosol occurs after PV is fully formed [37]. Therefore, as with *Toxoplasma gondii,* there may be two waves of effector secretion: one by rhoptry proteins during invasion and another one by dense granule and small granule proteins during parasite development.

More importantly, Muc25 is the first *Cryptosporidium* protein being shown to be exported into host microvilli, providing new insight into a potential mechanism of microvillar modulation by the parasite. Diarrheal diseases resulting from enteric infections may be associated with impaired epithelial barrier function [43]. Cryptosporidiosis is a leading cause of diarrhea in children and young animals [44]. In addition, *Cryptosporidium* infection causes pathological changes in the small intestine, including cell death, alterations in tight and adherens junctions, and loss of the microvillar brush border [45,46]. On the other hand, during parasite invasion, the attached sporozoites are engulfed by host cells, and the adjacent microvilli undergo elongation [47]. In this study, we demonstrate that Muc25 is secreted into host cell microvilli and may therefore be involved in *Cryptosporidium*-associated microvillar remodeling. However, the underlying mechanism of *Cryptosporidium*-mediated host microvillar damage remains unknown.

Although Muc25 export into host microvilli is not critical for parasite survival, it is likely to be involved in the pathogenesis of *C. parvum*. In this study, we were able to directly knock out the *Muc25* gene in *C. parvum*. Although the gene deletion reduced parasite growth *in vitro*, we did not observe significant differences in parasite load between GKO mice infected with *ΔMuc25* and Muc25-3HA. However, this could be due to the highly virulent nature of the *C. parvum* strain used, which induces extremely high parasite loads and mortality in GKO mice. The longer survival of GKO mice infected with *ΔMuc25* compared to those infected with Muc25-3HA also supports the role of Muc25 in *C. parvum* pathogenesis. Mechanistically, transcriptomic analysis revealed that the host genes associated with adherens junctions and amino acid metabolism were downregulated in *ΔMuc25*-infected HCT-8 cells compared to Muc25-3HA-infected cells. Previous studies have shown that *C. parvum* infection can lead to the disruption of barrier function through the downregulation of epithelial tight junctions and adherens junctions [45,48,49]. The disorganization of cellular junctions reduces intestinal epithelial integrity [50], which is a major cause of diarrhea during enteric infections [50,51]. However, the underlying mechanism of Muc25 in *C. parvum* pathogenesis remains to be elucidated. Further biological investigations into the mechanism of action of Muc25 should be conducted.

**Acknowledgements**

We thank Jilei Huang of the Instrumental Analysis & Research Center, South China Agricultural University for technical assistance in electron microscopy.

**Disclosure statement**

The authors have declared that no competing interests exist.

**Funding**

This work was supported by grants from the National Natural Science Foundation of China (32030109 and 32473052), Guangdong Major Project of Basic and Applied Basic Research (2020B0301030007), 111 Project (D20008), and Specific University Discipline Construction Project (2023B10564003).

**Author contributions**

NL and LX designed and supervised the research. JL, WW, ZY, SZ, FY and TH performed experiments. RX, JL, YG and YF analyzed data. RX and JL wrote the paper. LX and NL revised the draft. All authors have read and approved the final version of the manuscript.

**Data availability statement**

All data are found in the manuscript or supplemental materials. The RNA-seq data was deposited at NCBI under the BioProject number PRJNA1231048. The whole genomic sequencing data of *Muc25ΔSP* and *ΔMuc25* strain were deposited under the BioProject number PRJNA1261239. The raw numerical data in the manuscript are openly available in Mendeley Data (https://doi.org/ 10.17632/bk57yxws9v.1).

**References**

[1] Kotloff KL, Nasrin D, Blackwelder WC et al. The incidence, aetiology, and adverse clinical consequences of less severe diarrhoeal episodes among infants and children residing in low-income and middle-income countries: a 12-month case-control study as a follow-on to the Global Enteric Multicenter Study (GEMS). The Lancet Global Health. 2019; 7(5), e568-e584. doi: 10.1016/s2214-109x(19)30076-2

[2] Levine MM, Nasrin D, Acacio S et al. Diarrhoeal disease and subsequent risk of death in infants and children residing in low-income and middle-income countries: analysis of the GEMS case-control study and 12-month GEMS-1A follow-on study. Lancet Glob Health. 2020; 8(2), e204-e214. doi: 10.1016/S2214-109X(19)30541-8

[3] Gilbert IH, Vinayak S, Striepen B et al. Safe and effective treatments are needed for cryptosporidiosis, a truly neglected tropical disease. BMJ Glob Health. 2023; 8(8), e012540. doi: 10.1136/bmjgh-2023-012540

[4] Checkley W, White AC, Jr., Jaganath D et al. A review of the global burden, novel diagnostics, therapeutics, and vaccine targets for *Cryptosporidium*. Lancet Infect Dis. 2015; 15(1), 85-94. doi: 10.1016/S1473-3099(14)70772-8

[5] Timmermans M, Hubers W, Schroer D et al. The first commercially approved efficacious *Cryptosporidium* vaccine protecting New-Born calves from severe diarrhea. Veterinary Vaccine. 2024; 3(1), 100054. doi: 10.1016/j.vetvac.2024.100054

[6] Elliott DA & Clark DP. *Cryptosporidium parvum* induces host cell actin accumulation at the host-parasite interface. Infect Immun. 2000; 68(4), 2315-2322. doi: 10.1128/IAI.68.4.2315-2322.2000

[7] Borowski H, Thompson RC, Armstrong T et al. Morphological characterization of *Cryptosporidium parvum* life-cycle stages in an *in vitro* model system. Parasitology. 2010; 137(1), 13-26. doi: 10.1017/S0031182009990837

[8] Xu R, Beatty WL, Greigert V et al. Multiple pathways for glucose phosphate transport and utilization support growth of *Cryptosporidium parvum*. Nat Commun. 2024; 15(1), 380. doi: 10.1038/s41467-024-44696-3

[9] English ED, Guerin A, Tandel J et al. Live imaging of the *Cryptosporidium parvum* life cycle reveals direct development of male and female gametes from type I meronts. PLoS Biol. 2022; 20(4), e3001604. doi: 10.1371/journal.pbio.3001604

[10] Wallbank BA, Pardy RD, Brodsky IE et al. *Cryptosporidium* impacts epithelial turnover and is resistant to induced death of the host cell. mBio. 2024, e0172024. doi: 10.1128/mbio.01720-24

[11] Abdou AG, Harba NM, Afifi AF et al. Assessment of *Cryptosporidium parvum* infection in immunocompetent and immunocompromised mice and its role in triggering intestinal dysplasia. Int J Infect Dis. 2013; 17(8), e593-600. doi: 10.1016/j.ijid.2012.11.023

[12] Pardy RD, Wallbank BA, Striepen B et al. Immunity to *Cryptosporidium*: insights into principles of enteric responses to infection. Nat Rev Immunol. 2024; 24(2), 142-155. doi: 10.1038/s41577-023-00932-3

[13] Bouzid M, Hunter PR, Chalmers RM et al. *Cryptosporidium* pathogenicity and virulence. Clin Microbiol Rev. 2013; 26(1), 115-134. doi: 10.1128/CMR.00076-12

[14] Cevallos AM, Bhat N, Verdon R et al. Mediation of *Cryptosporidium parvum* infection in vitro by mucin-like glycoproteins defined by a neutralizing monoclonal antibody. Infect Immun. 2000; 68(9), 5167-5175. doi:

[15] Barnes DA, Bonnin A, Huang JX et al. A novel multi-domain mucin-like glycoprotein of *Cryptosporidium parvum* mediates invasion. Mol Biochem Parasitol. 1998; 96(1-2), 93-110. doi: 10.1016/s0166-6851(98)00119-4

[16] Van Klinken BJ, Dekker J, Buller HA et al. Mucin gene structure and expression: protection vs. adhesion. Am J Physiol. 1995; 269(5 Pt 1), G613-627. doi: 10.1152/ajpgi.1995.269.5.G613

[17] Puiu D, Enomoto S, Buck GA et al. CryptoDB: the *Cryptosporidium* genome resource. Nucleic Acids Res. 2004; 32(Database issue), D329-331. doi: 10.1093/nar/gkh050

[18] Xu Z, Guo Y, Roellig DM et al. Comparative analysis reveals conservation in genome organization among intestinal *Cryptosporidium* species and sequence divergence in potential secreted pathogenesis determinants among major human-infecting species. BMC Genomics. 2019; 20(1), 406. doi: 10.1186/s12864-019-5788-9

[19] Xu Z, Li N, Guo Y et al. Comparative genomic analysis of three intestinal species reveals reductions in secreted pathogenesis determinants in bovine-specific and non-pathogenic *Cryptosporidium* species. Microb Genom. 2020; 6(6), e000379. doi: 10.1099/mgen.0.000379

[20] Petersen C, Gut J, Doyle PS et al. Characterization of a > 900,000-M(r) *Cryptosporidium parvum* sporozoite glycoprotein recognized by protective hyperimmune bovine colostral immunoglobulin. Infect Immun. 1992; 60(12), 5132-5138. doi:

[21] Cevallos AM, Zhang X, Waldor MK et al. Molecular cloning and expression of a gene encoding *Cryptosporidium parvum* glycoproteins gp40 and gp15. Infect Immun. 2000; 68(7), 4108-4116. doi: 10.1128/iai.68.7.4108-4116.2000

[22] Li M, Yang F, Hou T et al. Variant surface protein GP60 contributes to host infectivity of *Cryptosporidium parvum*. Commun Biol. 2024; 7(1), 1175. doi: 10.1038/s42003-024-06885-0

[23] O'Connor RM, Burns PB, Ha-Ngoc T et al. Polymorphic mucin antigens CpMuc4 and CpMuc5 are integral to *Cryptosporidium parvum* infection in vitro. Eukaryot Cell. 2009; 8(4), 461-469. doi: 10.1128/EC.00305-08

[24] Akey ME, Xu R, Ravindran S et al. Apical secretory glycoprotein complex contributes to cell attachment and entry by *Cryptosporidium parvum*. mBio. 2023; 14(1), e0306422. doi: 10.1128/mbio.03064-22

[25] Gilchrist CA, Campo JJ, Pablo JV et al. Specific *Cryptosporidium* antigens associate with reinfection immunity and protection from cryptosporidiosis. J Clin Invest. 2023; 133(16), e166814. doi: 10.1172/JCI166814

[26] Feng Y, Torres E, Li N et al. Population genetic characterisation of dominant *Cryptosporidium parvum* subtype IIaA15G2R1. Int J Parasitol. 2013; 43(14), 1141-1147. doi: 10.1016/j.ijpara.2013.09.002

[27] Feng Y, Li N, Roellig DM et al. Comparative genomic analysis of the IId subtype family of *Cryptosporidium parvum*. Int J Parasitol. 2017; 47(5), 281-290. doi: 10.1016/j.ijpara.2016.12.002

[28] Wang T, Guo Y, Roellig DM et al. Sympatric recombination in zoonotic *Cryptosporidium* leads to emergence of populations with modified host preference. Mol Biol Evol. 2022; 39(7), msac150. doi: 10.1093/molbev/msac150

[29] Arrowood MJ. *Cryptosporidium* oocyst purification using discontinuous gradient centrifugation. Methods Mol Biol. 2020; 2052(43-59. doi: 10.1007/978-1-4939-9748-0_4

[30] Xu R, Feng Y, Xiao L et al. Insulinase-like protease 1 contributes to macrogamont formation in *Cryptosporidium parvum*. mBio. 2021; 12(2), e03405-03420. doi: 10.1128/mBio.03405-20

[31] Vinayak S, Pawlowic MC, Sateriale A et al. Genetic modification of the diarrhoeal pathogen *Cryptosporidium parvum*. Nature. 2015; 523(7561), 477-480. doi: 10.1038/nature14651

[32] Dos Santos Pacheco N & Soldati-Favre D. Coupling auxin-inducible degron system with ultrastructure expansion microscopy to accelerate the discovery of gene function in *Toxoplasma gondii*. Methods Mol Biol. 2021; 2369(121-137. doi: 10.1007/978-1-0716-1681-9_8

[33] Guerin A, Strelau KM, Barylyuk K et al. *Cryptosporidium* uses multiple distinct secretory organelles to interact with and modify its host cell. Cell Host Microbe. 2023; 31(4), 650-664.e656. doi: 10.1016/j.chom.2023.03.001

[34] Tandel J, English ED, Sateriale A et al. Life cycle progression and sexual development of the apicomplexan parasite *Cryptosporidium parvum*. Nat Microbiol. 2019; 4(12), 2226-2236. doi: 10.1038/s41564-019-0539-x

[35] Guerin A & Striepen B. The biology of the intestinal intracellular parasite *Cryptosporidium*. Cell Host Microbe. 2020; 28(4), 509-515. doi: 10.1016/j.chom.2020.09.007

[36] Guerin A, Roy NH, Kugler EM et al. *Cryptosporidium* rhoptry effector protein ROP1 injected during invasion targets the host cytoskeletal modulator LMO7. Cell Host Microbe. 2021; 29(9), 1407-1420.e1405. doi: 10.1016/j.chom.2021.07.002

[37] Dumaine JE, Sateriale A, Gibson AR et al. The enteric pathogen *Cryptosporidium parvum* exports proteins into the cytosol of the infected host cell. Elife. 2021; 10(e70451. doi: 10.7554/eLife.70451

[38] Li X, Yin J, Wang D et al. The mucin-like, secretory type-I transmembrane glycoprotein GP900 in the apicomplexan *Cryptosporidium parvum* is cleaved in the secretory pathway and likely plays a lubrication role. Parasit Vectors. 2022; 15(1), 170. doi: 10.1186/s13071-022-05286-8

[39] Strong WB, Gut J & Nelson RG. Cloning and sequence analysis of a highly polymorphic *Cryptosporidium parvu*m gene encoding a 60-kilodalton glycoprotein and characterization of its 15- and 45-kilodalton zoite surface antigen products. Infect Immun. 2000; 68(7), 4117-4134. doi: 10.1128/IAI.68.7.4117-4134.2000

[40] Gubbels MJ & Duraisingh MT. Evolution of apicomplexan secretory organelles. Int J Parasitol. 2012; 42(12), 1071-1081. doi: 10.1016/j.ijpara.2012.09.009

[41] Wetzel DM, Schmidt J, Kuhlenschmidt MS et al. Gliding motility leads to active cellular invasion by *Cryptosporidium parvum* sporozoites. Infect Immun. 2005; 73(9), 5379-5387. doi: 10.1128/IAI.73.9.5379-5387.2005

[42] Gao X, Yin J, Wang D et al. Discovery of new microneme proteins in *Cryptosporidium parvum* and implication of the roles of a rhomboid membrane protein (CpROM1) in host-parasite interaction. Front Vet Sci. 2021; 8(778560. doi: 10.3389/fvets.2021.778560

[43] Guttman JA & Finlay BB. Tight junctions as targets of infectious agents. Biochim Biophys Acta. 2009; 1788(4), 832-841. doi: 10.1016/j.bbamem.2008.10.028

[44] Kotloff KL, Nataro JP, Blackwelder WC et al. Burden and aetiology of diarrhoeal disease in infants and young children in developing countries (the Global Enteric Multicenter Study, GEMS): a prospective, case-control study. Lancet. 2013; 382(9888), 209-222. doi: 10.1016/S0140-6736(13)60844-2

[45] Kumar A, Chatterjee I, Anbazhagan AN et al. *Cryptosporidium parvum* disrupts intestinal epithelial barrier function via altering expression of key tight junction and adherens junction proteins. Cell Microbiol. 2018; 20(6), e12830. doi: 10.1111/cmi.12830

[46] Luo C, Xu Y, Zhang J et al. *Cryptosporidium parvum* disrupts intestinal epithelial barrier in neonatal mice through downregulation of cell junction molecules. PLoS Negl Trop Dis. 2024; 18(5), e0012212. doi: 10.1371/journal.pntd.0012212

[47] Leitch GJ & He Q. Cryptosporidiosis-an overview. J Biomed Res. 2012; 25(1), 1-16. doi: 10.1016/S1674-8301(11)60001-8

[48] Greigert V, Saraav I, Son J et al. *Cryptosporidium* infection of human small intestinal epithelial cells induces type III interferon and impairs infectivity of Rotavirus. Gut Microbes. 2024; 16(1), 2297897. doi: 10.1080/19490976.2023.2297897

[49] Lamisere H, Bhalchandra S, Kane AV et al. Differential response to the course of *Cryptosporidium parvum* infection and its impact on epithelial integrity in differentiated versus undifferentiated human intestinal enteroids. Infect Immun. 2022; 90(11), e0039722. doi: 10.1128/iai.00397-22

[50] Chelakkot C, Ghim J & Ryu SH. Mechanisms regulating intestinal barrier integrity and its pathological implications. Exp Mol Med. 2018; 50(8), 1-9. doi: 10.1038/s12276-018-0126-x

[51] Groschwitz KR & Hogan SP. Intestinal barrier function: molecular regulation and disease pathogenesis. J Allergy Clin Immunol. 2009; 124(1), 3-20; quiz 21-22. doi: 10.1016/j.jaci.2009.05.038

**Figure Legends:**

**Figure 1. Expression characteristics of Muc25 in *Cryptosporidium parvum*.**

(**A**) Phylogenetic relationship of the *Muc25* gene in *Cryptosporidium* spp. The tree was constructed using the maximum likelihood method and the Tamura-Nei model with 1,000 replicates for bootstrapping. The subtypes and sample IDs of *Cryptosporidium* spp. are indicated. (**B**) Violin plots showing the relative expression of the *Muc25* gene in *C. parvum* sporozoites and developmental stages in HCT-8 cultures, as indicated by TPM values from RNA-seq analysis of the transcriptome. Each dot represents one gene, and some dense granule genes (DG1, cgd3_600; DG2, cgd5_1440; DG3, cgd1_590 and DG4, cgd3_1690) and small granule genes (SG1, cgd1_3810 and SG2, cgd4_3050) are used as controls. (**C**) Schematic of endogenous tagging of the *Muc25* gene with 3 copies of the HA epitope tag. The position of the single guide RNA (sgRNA) in the 3’UTR of target gene and the template for homologous recombination are shown. Nluc, nanoluciferase; Neo, neomycin resistance marker; *pEno*, enolase promoter. (**D**) Confirmation of the correct gene modification in the Muc25-3HA tagging strain by PCR. Fecal genomic DNA extracted from wild type (WT) and Muc25-3HA were used for PCR. Primer positions for 5’ insertion (5’ ins) and 3’ insertion (3’ ins) verification are shown in (C), and primer sequences are provided in Table S1. (**E**) Immunofluorescence of Muc25-3HA in sporozoites. Transgenic sporozoites were fixed and stained with rabbit anti-HA antibody (red), the nuclear stain Hoechst (blue), and *Vicia villosa* lectin (VVL, green), which recognizes *C. parvum*, and. Scale bars = 2 μm. (**F**) Ultrastructure expansion microscopy (U-ExM) of Muc25-3HA in sporozoites. Transgenic sporozoites were fixed, expanded in gel and stained with rat anti-HA (green), Hoechst (blue), and NHS-ester, which stains secretory organelles (red). Scale bars = 5 μm.

**Figure 2. Export of Muc25 into host cell microvilli during *C. parvum* development.**

(**A**) Immunofluorescence detection of Muc25-3HA in intracellular parasites grown in HCT-8 cells. Cells were fixed at 48 h post infection (hpi) and stained with rabbit anti-HA antibody (red), VVL (green), and Hoechst (blue). Scale bars = 2 μm. (**B**) Immunofluorescence analysis of Muc25-3HA secretion into host cells during parasite invasion and early development at different time points. HCT-8 cells were infected with Muc25-3HA parasites, fixed from 30 min to 2.5 hpi, and stained with rabbit anti-HA antibody (red), VVL (green), and Hoechst (blue). Scale bars = 2 μm. (**C**) Immunoelectron microscopy micrographs of Muc25-3HA parasites. Mouse ileum infected with Muc25-3HA parasites was fixed and stained with rabbit anti-HA followed by goat anti-rabbit IgG conjugated 10-nm colloidal gold. Scale bars = 500 nm. Gold particles are seen on host cell microvilli.

**Figure 3. Involvement of the signal peptide in the translocation of Muc25.**

(**A**) Schematic of the knockout of the signal peptide sequence of the *Muc25* gene in *C. parvum*. The position of the single guide RNA (sgRNA) in the signal peptide (SP) of the target gene and the template for homologous recombination are shown. Nluc, nanoluciferase; Neo, neomycin resistance marker; *pEno*, enolase promoter. (**B**) Confirmation of the correct gene modification in *Muc25ΔSP* strain by PCR. Fecal genomic DNA extracted from WT and *Muc25ΔSP* parasites was used for PCR. Primer positions for 5’ ins and 3’ ins verification are indicated in (A) and primer sequences are provided in Table S1. (**C**) Alignment of reads from whole genome sequencing of the *Muc25ΔSP* strain at the site of the SP deletion. The coverage depths of 5'UTR and signal peptide are shown in the figure. (**D**) Immunofluorescence analysis of Muc25ΔSP in sporozoites. Transgenic sporozoites were fixed and stained with rabbit anti-HA antibody (red), VVL (green), and Hoechst (blue). Scale bars = 2 μm. (**E**) Immunofluorescence analysis of Muc25-3HA and Muc25ΔSP in intracellular parasites grown in HCT-8 cells. Cells were fixed at 48 hpi and stained with rabbit anti-HA antibody (red), VVL (green), and Hoechst (blue). Scale bars = 2 μm.

**Figure 4. Involvement of Muc25 in growth and pathogenicity of *C. parvum*.**

(**A**) Schematic of the replacement of the *Muc25* gene with a Nluc-Neo cassette. The position of the sgRNA in the middle of the target gene and the template for homologous recombination are shown. Nluc, nanoluciferase; Neo, neomycin resistance marker; *pEno*, enolase promoter. (**B**) Confirmation of the correct genetic modification in the *ΔMuc25* strain. Fecal genomic DNA extracted from WT and *ΔMuc25* parasites was used for PCR. Primer positions for 5’ ins and 3’ ins verification are shown in (A), and primer sequences are provided in Table S1. (**C**) Alignment of reads from whole genome sequencing of the *ΔMuc25* strain at the site of the gene deletion. The coverage depths of 5'UTR and open reading frame are shown in the figure. (**D**) Growth of Muc25-3HA and *ΔMuc25* strains in HCT-8 cells. Relative luminescence values of transgenic parasites were quantified at the indicated time points. Values are presented as means ± SEM. *P* values from two-way ANOVA with Sidak’s multiple comparison are shown; ns, not significant. (**E**) Parasite loads of Muc25-3HA and *ΔMuc25* strains in GKO mice. Each mouse was infected with 1 × 10^3^ oocysts. Relative luminescence values from collected feces were measured at indicated days post infection. Each dot represents mean ± SD of an infection study performed with 6 mice per group (each mouse was housed individually). No significant difference in parasite load was observed between Muc25-3HA and *ΔMuc25* strains according to the two-way ANOVA with Sidak’s multiple comparison of data. (**F**) Body weight of GKO mice infected with Muc25-3HA and *ΔMuc25* parasites. (**G**) Comparison of the area under the curve (AUC) of the body weight data. The difference between the two groups was significant by two-tailed Mann-Whitney U test; ns, not significant. (**H**) Survival curve for GKO mice infected with Muc25-3HA and *ΔMuc25* parasites. *P* value from the log-rank Mantel-Cox test is shown.

**Figure 5. Transcriptomic changes of host genes after the ablation of Muc25.**

(**A**) Relative transcription of *Muc25* in HCT-8 cells infected with Muc25-3HA and *ΔMuc25* strain. Data were extracted from RNA-seq at 12 hpi and are presented as mean ± SD. *P* value from two-tailed Mann-Whitney U test is shown. (**B**) Volcano plot of differentially expressed genes (DEGs) in HCT-8 cells infected with Muc25-3HA and *ΔMuc25* parasites at 12 hpi. Upregulated genes are shown in red with fold change of gene transcript level in *ΔMuc25* vs Muc25-3HA > 1.5 and padj < 0.05. Downregulated genes are shown in blue with fold change of gene transcript level in *ΔMuc25* vs Muc25-3HA < -1.5 and padj < 0.05. (**C**) GO enrichment analysis of downregulated genes of HCT8 cells infected with *ΔMuc25* compared to cells infected with Muc25-3HA at 12 hpi, including biological process (BP), cellular component (CC), molecular function (MF), and Kyoto Encyclopedia of Genes and Genomes (KEGG). (**D**) GO enrichment analysis of upregulated genes of HCT8 cells infected with *ΔMuc25* compared to cells infected with Muc25-3HA at 12 hpi. (**E**) Venn diagram of enriched GO terms shared by different groups. (**F**) GO enrichment analysis of genes that were downregulated in the *ΔMuc25* group compared to the Muc25-3HA group but upregulated in the Muc25-3HA group compared to the control group based on the Venn analysis.

**Supplementary material**

**Table S1 Primers and plasmids used in the study.**

**Figure S1 Immunofluorescence of Muc25-3HA in sporozoites.**

Transgenic sporozoites were fixed and stained with rabbit anti-HA antibody (red), the nuclear stain Hoechst (blue), and *Vicia villosa* lectin (VVL, green), which recognizes *C. parvum*, and. Scale bars = 5 μm.

**Figure S2 Ultrastructure expansion microscopy (U-ExM) of Muc25-3HA in sporozoites.**

Transgenic sporozoites were fixed, expanded in gel and stained with rat anti-HA (green), Hoechst (blue), and NHS-ester, which stains secretory organelles (red). Scale bars = 5 μm.

**Figure S3 Immunofluorescence detection of Muc25-3HA in intracellular parasites.**

(**A**) Immunofluorescence of Muc25-3HA in the trophozoites and meronts (4 nuclei and 8 nuclei). HCT-8 cells were infected with the Muc25-3HA strain, fixed at 24 h post infection (hpi) and stained with rabbit anti-HA antibody (red), VVL (green), and Hoechst (blue). Scale bars = 2 μm. (**B**) Immunofluorescence of Muc25-3HA in the female gametes and male gamonts. HCT-8 cells were infected with the Muc25-3HA strain, fixed at 48 h post infection (hpi) and stained with rabbit anti-HA antibody (red), anti-AP2-F2 that recognizes the nucleus of female gametes (green), and Hoechst (blue). Scale bars = 2 μm.
